# Exploring Integrative Approaches: Insights Into Complementary Medicine Practices Among Breast Cancer Survivors in Saudi Arabia

**DOI:** 10.7759/cureus.52282

**Published:** 2024-01-15

**Authors:** Atlal Abusanad, Reem Ujaimi, Marah A Alotaibi, Lama A Alharbi, Nouf Alatawi, Abeer A Algarni, Ali Samkari

**Affiliations:** 1 Faculty of Medicine, King Abdulaziz University (KAUH), Jeddah, SAU; 2 Surgery, Faculty of Medicine, King Abdulaziz University (KAUH), Jeddah, SAU

**Keywords:** cam, side effects, benefits, practices, survivors, breast cancer, alternative medicine, complementary medicine

## Abstract

Objectives: Complementary and integrative medicine (CAM) is a prevalent approach often used with conventional medical practices The study aims to understand the factors influencing breast cancer (BC) survivors' decisions regarding CAM therapy and the drivers behind their use.

Setting: This research was conducted at an academic hospital in Saudi Arabia. The study used cross-sectional research with a questionnaire. Participants were enrolled in the study through BC survivors' groups on WhatsApp. Individuals who were actively following up at the clinic were also interviewed. Informed consent was obtained.

Results: The study surveyed 211 BC survivors, aged 34-49 (50.2%), who had undergone surgery (93.4%), hormonal therapy (66.4%), and chemotherapy (87.7%). Less than half (44.5%) had chronic conditions such as diabetes and hypertension. CAM users were 43.6%. The most used CAM modalities were Zamzam water, honey, and water read-upon Quran. A significant motivator for CAM use was to boost the immune system. More than half of patients used less than 100 SAR per month on CAM modalities. Exactly 80.4% of CAM users perceived benefits from CAM use. Predictors of CAM use included higher family monthly income, radiation therapy, and being diagnosed from 1 to 5 years. BC survivors without medical conditions after diagnosis were less likely to use CAM.

Conclusion: The study highlights the prevalence, predictive factors, motivations, and perceived benefits of CAM use among BC survivors in Saudi Arabia, emphasizing the need for understanding and integration into cancer care plans and the need for further research on CAM safety and efficacy.

## Introduction

Breast cancer (BC) is the second leading cause of cancer-related deaths and the most common cancer among women in Saudi Arabia, with incidence and mortality rates of 14.2% and 8.4%, respectively [1]. Globally, it led to 685,000 deaths in 2020, with an incidence rate of 2.3 million among women. BC is the most common cancer globally; as of the end of 2020, 7.8 million women were diagnosed within the previous five years. BC affects women at any age [2]. The incidence, however, rises with age.

The Saudi National Cancer Registry reported that 2,459 women were diagnosed with BC in 2020. The region with the highest incidence rate was the eastern region, followed by Riyadh, and 51 years old was the median age of diagnosis [1,3]. A previously published paper reporting the incidence of BC among Saudi women from 2001 to 2017 reported an increase in the incidence of 351.9%, contributing to the overall fast-rising burden of BC. Further, the percentage of Saudi women with BC increased by 55%, from 19.9% to account for 30.9% of all malignancies among Saudi females [4]. The common risk factors of BC include abnormal menstrual history, the use of oral contraceptives, family history, nulliparity, and obesity [5].

Complementary medicine is defined as non-mainstream practice often taken in addition to conventional medications by the National Center for Complementary and Integrative Health (NCCIH) [5,6]. The use of complementary and integrative medicine (CAM) varies across regions, ranging from 9.8% to 76%, and is partly determined by socioeconomics, geographical location, and religious beliefs [7,8].

CAM is categorized into five classes by the National Center for Complementary and Alternative Medicine, United States of America (USA), including alternative medical categories, biologically based therapies, mind-body interventions, manipulation and body-based methods, and energy therapies [9]. The use of yoga, meditation, and chiropractors among adults above 18 in the USA increased from 2012 to 2017. In 2012 and 2017, yoga was the supplemental health practice that US people employed the most (14.3%) [6]. Conversely, the most common types of CAM practiced in Saudi Arabia are associated with religious beliefs, such as the Holy Quran, black seed, Myrrh, honey, and cupping therapy [10-12]. Up to 10%-80% of cancer patients use CAM, such as prayers and nutritional supplements [13-17].

The common CAM used in the USA and Europe are acupuncture, massage therapy, and yoga. Conversely, the common CAM used in Saudi Arabia are Zamzam (holy water) and Ruqya (Quran recitation), cupping/hijama, drinking honey, camel milk, and urine, which are rooted in cultural and religious beliefs. Family members were the most frequent source of information about the use of CAM (81.6%) [18,19].

​​The annual expenditures on CAM are up to US$59 billion in the USA and £1.6 billion in the United Kingdom, which are significantly higher than that in Saudi Arabia. On the other hand, Saudi patients spend 650,000 USD on CAM visits and products [20,21]. The annual expenditure for CAM in Saudia Arabia was <1000 Saudi Arabian Riyal (SAR), equivalent to US$266.7 in 71.4% of patients and between 1000 SAR ($266.7) and 5,000 SAR (US$1333.3) in 28.6% of patients. However, the individual expenditure did not exceed 5,000 SAR (US$1333.3). Data are sparse regarding how much Saudis spend annually on CAM for cancer treatment. Most users start CAM before visiting a physician or knowing their diagnosis. Relatively poor access to the health system and the low cost of CAM may partly account for this [20].

Cancer patients may favor CAM, which they may consider "safe and natural," over radiation and chemotherapy. According to data already available, CAM is used by 50% of all cancer patients, and this usage has increased recently [21]. The Holy Quran, honey, Myrrh, black seed, and cupping therapy are some of the most popular CAMs in Saudi Arabia. Most CAM users started using it before seeing a doctor, and some may have done so before being aware of their illnesses. The duration of the procedure and difficulty gaining access to the healthcare system may be to blame for this practice. It might also be because CAM is widely accessible and inexpensive, particularly for everyday items such as honey and Nigella sativa. The importance of this practice stems from the potential inadvertent interactions between several CAM herbs and oils and various anti-cancer medications. Cancer patients in Saudi Arabia frequently use camel products as CAM. These goods have brucellosis risk and have been connected to MERS [21].

The prevalence of CAM is high in the USA. In the past year, 62% of persons in the USA over the age of 18 utilized CAM, including prayer, for health concerns, according to data from the 2002 US National Health Interview Survey (NHIS) [22]. In order to lessen the negative effects of cancer treatment, there is an increasing demand for CAM among cancer patients [23]. Women with BC may use CAM to enhance their immunity, stop the progression of their disease, cure their condition, or improve their quality of life [24]. The majority of CAM users were female BC patients. CAM use was substantially correlated with monthly income, work status, and educational attainment. These findings are consistent with what has been reported from earlier research. Although there was no conclusive link between the stage of cancer and CAM use, the percentage of individuals with more advanced diseases who used CAM was higher [24]. The desire to use CAM is driven by pursuing remedies that align with the person’s beliefs, principles, and philosophy of well-being and life [25].

Cancer survivors use CAM for various reasons despite limited evidence of their efficacy. These include preventing and treating the side effects of conventional therapeutic approaches, increasing survival rates, preventing and controlling comorbidities, supporting overall health and well-being, and addressing issues poorly addressed by conventional medicine [26]. Cancer survivors who desire non-pharmacological treatments for their symptoms may also turn to CAM [27,28]. Cancer survivors utilize various quantities and forms of CAMs. Most CAM users among cancer survivors, especially dietary supplements, are BC survivors.

CAM use is prevalent all around the world. Adult CAM utilization was common in Saudi Arabia, with prevalence rates ranging from 65% to 80% [29]. However, various CAM practices may exhibit multiple side effects and potential toxicities, and patients are frequently uninformed of these potential risks [30]. Research indicates that despite the great incidence of CAM usage, patients frequently do not disclose their utilization of CAM to their traditional healthcare physician [27].

## Materials and methods

Study design and participants: This cross-sectional study was conducted on adult females with BC from February to August 2023. We aimed to include a sample size of 211 to obtain a 95% confidence interval (95%CI) with a 2% margin of error. A survey was distributed to BC survivor groups on WhatsApp, and individuals closely tracking their recovery at the clinic were also incorporated into this research.

Research ethics: Informed consent to participate in this study was obtained online from each participant before data were collected. Ethical approval was obtained from the College of Medicine Ethical Committee at King Abdulaziz University, Jeddah, Saudi Arabia (Reference No. 70-23).

Data collection: We used a cross-sectional design with a structured questionnaire to collect participant data. The questionnaire covered demographic information, medical history, CAM usage, motivations, economic aspects, and perceived benefits or side effects. The data were anonymized and analyzed to conclude CAM patterns among BC patients.

We investigated diverse demographic characteristics, such as age category, the area of their residence, marital status, education level, employment status, family monthly income, and smoking status.

Participants were also required to provide information on medical aspects, including their cancer stage (non-metastatic, breast and lymph nodes, or metastatic), time since diagnosis (less than a year, one to five years, or more than five years), and type of treatment. Participants could choose multiple options (such as surgery, radiation, chemotherapy, hormonal, and combination) if they have a family history of BC or no comorbidities (such as diabetes mellitus, hypertension, and hyperlipidemia). Participants indicated the presence of multiple conditions, including cardiovascular, respiratory, inflammatory disease, other medical conditions, or none of the above. If they have psychological conditions, such as anxiety or depression if yes, participants specified if they were taking psych medication.

The survey focused on the participants' use of CAM, by asking if they have ever used it or not, initiation of CAM use (before treatment, during treatment, after treatment, or not using CAM), and types of CAM used. Participants could select from various CAM options, including dietary supplements, traditional remedies, and lifestyle practices, and what motivated them to start implementing CAM into their lifestyle.

Respondents were also asked about their monthly expenditure on CAM, categorized as less than 100 riyals, 100-500 riyals, or more than 500 riyals). Participants were asked to disclose how they were introduced to CAM, with options including friends/family, personal experience, lectures/courses, books/online articles, cancer organizations, and health providers. They shared whether they perceived benefits from CAM, and if so, the specific improvements noticed in physical health, mental health, and general well-being. They also reported any undesirable effects experienced due to CAM, choosing from a list of potential side effects.

Statistical analysis: Statistical analysis was done using RStudio (R version 4.3.0; R Development Core Team, Vienna, Austria). We used frequencies and percentages to express variables. A multiple-response analysis was employed to assess participants’ responses to variables with multiple valid responses (treatment received, chronic conditions, medical conditions after cancer diagnosis, motivators for CAM use, sources of information, CAM benefits, and CAM-related undesirable effects). The predictors of CAM use were assessed by applying a multivariable logistic regression analysis using a binary variable as a dependent variable (CAM use). The following independent variables were considered in the initial modeling: age, nationality, city, marital status, educational level, employment status, family monthly income, smoking, cancer stage, time since diagnosis, treatment received, chronic conditions, medical conditions after cancer diagnosis, and having anxiety/depression after cancer diagnosis. Variables were selected based on a stepwise backward selection method, and the finally retained variables were exclusively presented in the respective table. Results were expressed as odds ratio (OR) and 95%CI. Statistical significance was considered at p < 0.05.

## Results

Demographic characteristics of patients: Most participants fell within the age group of 34-49 years, constituting 106 (50.2%) of the sample. Saudi nationals comprised the largest cohort, accounting for 156 (73.9%) of respondents. In terms of city of residence, the highest frequency was observed in Jeddah, with 116 (55.0%) of participants residing there. Most respondents were married, representing 174 (82.5%) of the sample. Regarding educational attainment, 102 (48.3%) of participants held a bachelor's degree or higher. In terms of employment status, a significant proportion (123, 58.3%) reported being unemployed. Regarding family monthly income, the majority (140, 66.4%) reported an income of less than 14,000 SAR. Furthermore, most respondents had never smoked, with 192 (91.0%) reporting no history of smoking, while current smokers and former smokers constituted five (2.4%) and 14 (6.6%) of the participants, respectively (Table 1).

**Table 1:** Demographic characteristics of participants (n=211) (%) The data have been represented as N.

Characteristics	N (%)
Age (years)	
18 to 33	12 (5.7%)
34 to 49	106 (50.2%)
50 to 65	89 (42.2%)
66 or more	4 (1.9%)
Nationality	
Saudi	156 (73.9%)
Non-Saudi	55 (26.1%)
City	
Jeddah	116 (55.0%)
Riyadh	12 (5.7%)
Other	83 (39.3%)
Marital status	
Single	14 (6.6%)
Married	174 (82.5%)
Divorced/ Widowed	23 (10.9%)
Educational level	
Illiterate	3 (1.4%)
Elementary school	8 (3.8%)
Intermediate school	13 (6.2%)
High school	56 (26.5%)
Diploma	29 (13.7%)
Bachelor's degree or higher	102 (48.3%)
Employment status	
Unemployed	123 (58.3%)
Employed	57 (27.0%)
Retired	31 (14.7%)
Family monthly income (SAR)	
<14,000	140 (66.4%)
14,000 or more	71 (33.6%)
Smoking	
Never smoked	192 (91.0%)
Current	5 (2.4%)
Former	14 (6.6%)

**Figure 1:**
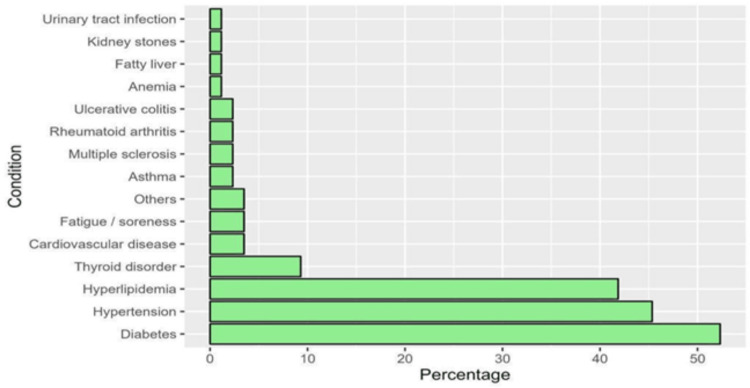
The percentages of patients with chronic diseases (n=86)

Clinical characteristics of patients: Most patients were at the non-metastatic stage of cancer, accounting for 197 (93.4%). Moreover, a substantial proportion of participants (125, 59.2%) were diagnosed with BC within the last one to five years. Notably, a significant number (197, 93.4%) had undergone surgery as part of their treatment regimen. Additionally, 140 (66.4%) had received hormonal therapy, and 185 (87.7%) had undergone chemotherapy. Less than half of the patients had a history of chronic conditions (94, 44.5%), with diabetes and hypertension prevalent among 52.3% and 45.3% of them, respectively (Figure 1).

Regarding the presence of medical conditions after cancer diagnosis, 94 (44.5%) reported experiencing such conditions, with bone disease being the most prevalent at 58 (61.7%) (Table 2).

**Table 2:** Clinical characteristics of participants (n=211) *Descriptive statistics are based on 46 patients who had been diagnosed with anxiety/depression after cancer diagnosis. ¥Descriptive statistics are based on 94 patients who had medical conditions after cancer diagnosis. (%) The data have been represented as N.

Characteristics	N=211
Cancer stage	
Only breast	88 (41.7%)
Breast and lymph nodes	109 (51.7%)
Metastatic	14 (6.6%)
Time since diagnosis	
Less than a year	39 (18.5%)
Between 1-5 years	125 (59.2%)
More than 5 years	47 (22.3%)
Family history of cancer (first-degree relatives)	45 (21.3%)
Diagnosed with anxiety/depression after cancer diagnosis	46 (21.8%)
Taking any psych medications*	25 (12%)
Treatment received	
Chemotherapy	185 (87.7%)
Endocrine therapy	140 (66.4%)
Radiotherapy	172 (81.5%)
Surgery	197 (93.4%)
Chronic conditions	86 (40.8%)
Had medical conditions after cancer diagnosis	94 (44.5%)
Types of medical conditions after cancer diagnosis¥	
Bone disease	58 (61.7%)
Nervous system disorder	24 (25.5%)
Cardiovascular	16 (17.0%)
Inflammatory disease	39 (41.5%)
Respiratory	13 (13.8%)
Others	7 (7.4%)

**Figure 2:**
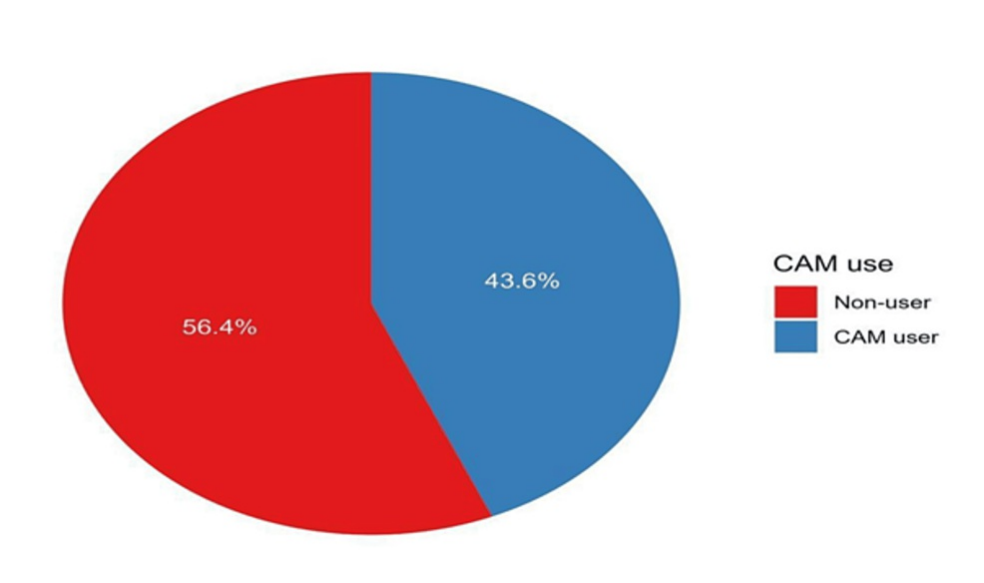
The prevalence of CAM use among patients under study (n=211)

Patterns of CAM use: We identified a total of 92 patients with CAM use (43.6%). Most CAM users began their CAM therapy during their cancer treatment, accounting for 38 (41.8%). The most used CAM modalities were Zamzam water (80.4%), honey (65.2%), and water read-upon Quran (63.0%) (Figure 2).

Furthermore, a significant motivator for CAM use among these patients was to boost their immune system, with 62 (67.4%) of CAM users reporting this as a reason. Additionally, a substantial proportion (70, 76.1%) relied on information from friends and family members as their primary source of information about CAM. Notably, more than half of patients used to pay <100 SAR per month for CAM modalities (Table 3).

**Table 3:** Patterns of CAM use (n=92) (%) The data have been represented as N.

Characteristics	N (%)
Time since starting CAM use	
Before start treatment	32 (35.2%)
During treatment	38 (41.8%)
After treatment	21 (23.1%)
Motivator of using CAM	
It causes no harm	43 (46.7%)
To decrease the risk of cancer recurrence	35 (38.0%)
It might strengthen the immunity	62 (67.4%)
To improve mental wellbeing	35 (38.0%)
To improve sleep	15 (16.3%)
To relieve side effects of cancer treatments	37 (40.2%)
Expenditure on CAM methods per month (SAR)	
<100	47 (51.1%)
100 to 500	34 (37.0%)
>500	11 (12.0%)
Sources of information about CAM	
Books/Online articles	21 (22.8%)
Health providers	2 (2.2%)
CAM therapists	11 (12.0%)
Cancer organizations	4 (4.3%)
Friends/family members	70 (76.1%)
Lectures/courses	6 (6.5%)
Own experience and interest	37 (40.2%)

Among the participants, 74 (80.4%) of CAM users perceived benefits from CAM use. The most reported benefits included improvements in mental health and general well-being, with 49 (66.2%) for each. In contrast, 17 (18.5%) reported undesirable effects due to CAM use. The undesirable effects included increased urination (8, 47.1%), nausea (7, 41.2%), and pain (5, 29.4%) (Table 4).

**Table 4:** Participants’ experiences due to CAM use (n=92) *Descriptive statistics are based on 74 responses from those who had encountered the benefits of CAM. ¥Descriptive statistics are based on 17 responses from those who had encountered undesirable effects due to CAM. (%) The data have been represented as N.

Characteristics	N (%)
Perceived benefits of CAM use	
No	18 (19.6%)
Yes	74 (80.4%)
Benefits*	
Improvement of mental health	49 (66.2%)
Improvement of the general wellbeing	49 (66.2%)
Improvement of physical health	40 (54.1%)
Perceived undesirable effects due to CAM use	
No	75 (81.5%)
Yes	17 (18.5%)
Types of encountered undesirable effects¥	
Bloating	3 (17.6%)
Constipation	4 (23.5%)
Diarrhea	3 (17.6%)
Stomach discomfort	2 (11.8%)
Pain	5 (29.4%)
Increased urination	8 (47.1%)
Nausea	7 (41.2%)
Fatigue/soreness	3 (17.6%)
Increased blood sugar	2 (11.8%)
Allergic reactions	1 (5.9%)
Fever	1 (5.9%)

Predictors of CAM use: Participants with a family monthly income of 14,000 SAR or more were 2.42 times more likely to use CAM (OR = 2.42, 95%CI: 1.25-4.78, p = 0.010) compared to those with incomes below 14,000 SAR. Additionally, individuals who had received radiation therapy as part of their treatment were significantly more likely to use CAM (OR = 7.27, 95%CI: 2.37-26.1, p = 0.001) than those who did not. CAM use was also predicted by being diagnosed in the previous five years (OR = 2.75, 95%CI: 1.10-7.43, p = 0.036). In contrast, BC survivors who had not experienced any medical conditions after their cancer diagnosis were less likely to use CAM (OR = 0.44, 95%CI: 0.21-0.89, p = 0.025) in comparison to those who had medical conditions post-diagnosis. CAM use was also less likely to be prevalent among patients who underwent chemotherapy (OR = 0.26, 95%CI: 0.08-0.74, p = 0.014), hormonal therapy (OR = 0.33, 95%CI: 0.15-0.66, p = 0.002) and surgery (OR = 0.17, 95%CI: 0.03-0.87, p = 0.025) (Table 5).

**Table 5:** Predictors of CAM use OR = Odds ratio, CI = Confidence interval p-value considered significant = p < 0.05

Characteristics	OR	95% CI	p-value
Family monthly income (SAR)			
<14,000	Reference	Reference	
14,000 or more	2.42	1.25, 4.78	0.010
Smoking			
Never smoked	Reference	Reference	
Current	0.16	0.01, 1.20	0.114
Former	0.35	0.05, 1.64	0.222
Time since diagnosis			
Less than a year	Reference	Reference	
Between 1-5 years	2.75	1.10, 7.43	0.036
More than 5 years	1.94	0.64, 6.18	0.249
Treatment received			
Chemotherapy	0.26	0.08, 0.74	0.014
Endocrine therapy	0.33	0.15, 0.66	0.002
Radiotherapy	7.27	2.37, 26.1	0.001
Surgery	0.17	0.03, 0.87	0.037
Had medical conditions after cancer diagnosis			
Nervous system disorder	2.55	0.88, 7.90	0.091
None	0.44	0.21, 0.89	0.025

## Discussion

​​We investigated the frequency, patterns, influencing factors, and perceived benefits of CAM among BC survivors in Saudi Arabia. We also explored the source of information and the cost of CAM use. Our study demonstrated that most of the participants had a non-metastatic stage, were diagnosed within the last one to five years, and had undergone surgical and chemotherapy treatments. Many had a history of chronic conditions, with bone disease being the most prevalent. About 44% of the participants incorporated CAM into their cancer treatment, spending less than 100 SAR per month on CAM modalities. Factors influencing CAM use included higher family income, chemotherapy, endocrine therapy, and radiotherapy plus post-diagnosis medical conditions. Almost two-thirds of the cohort had been diagnosed with BC within the last one to five years. A history of chronic diseases was found in less than half of the participants (40.8%), with diabetes and hypertension accounting for 52.3% and 45.3%, respectively. Regarding the presence of medical conditions after cancer diagnosis, 44.5% reported experiencing such conditions, with bone disease being the most prevalent at 61.7%.​ 

Generally, a total of 92 patients were CAM users (43.6%, Figure 2). In a comparable study, 79% of cancer survivors in the USA reported taking one or more CAM, in contrast to 68% of persons who did not suffer from cancer. When looking at the responses, the main finding is that 41.8% of CAM users started their CAM use during their cancer treatment [26,31]. A recently published study on the use of CAM among cancer patients demonstrated that 35% of their subjects admitted to using CAM after their diagnosis of cancer.

In addition, a significant proportion (76.1%) relied on information from friends and family members as the main source of information for CAM. According to a previous study to assess the knowledge and practice of residents in Riyadh city of CAM, family, friends, and relatives were the primary sources of CAM information for 46.3% of participants, whereas mass media (such as television, newspapers, and radio) were a source of knowledge and attitude for 46.5% of the research group.

In addition, one of the main reasons for these patients to use CAM was to improve their immunity. Most CAM users (67.4%) cite this as their primary reason for using CAM. In contrast, a study conducted in King Abdulaziz Medical City in Riyadh among cancer patients revealed that 75% of participants were using CAM to treat cancer.

In addition, our study found that 80.4% of CAM users reported perceiving benefits from its use. Similarly, a previous study revealed that 86% of CAM users believed it helped them and had a positive experience. Similarly, another study found that 74.3% of individuals experienced similar improvements in their mental state (56.8%), hunger (23.5%), and physical stamina (7.4%) [32,33].

Participants with a family monthly income of 14,000 SAR or more were 2.42 times more likely to use CAM (OR = 2.42, 95%CI: 1.25-4.78, p = 0.010) than those with incomes below 14,000 SAR. This is relevant to a previous study, which showed CAM consumers appeared to be more well-educated and middle-aged. They also discovered a correlation between higher financial status and increased CAM use. The financial burden of cancer treatment and the additional costs associated with CAM could contribute to financial difficulties [26]. According to our study, smokers were less likely to use CAM than non-smokers (OR = 0.16, 95%CI: 0.01-1.20, p = 0.114), and this finding relates to a similar study showing using CAM modalities was negatively correlated with smoking cigarettes [34].

CAM use was also less likely to be prevalent among patients with chemotherapy (OR = 0.26, 95%CI: 0.08-0.74, p = 0.014). Perhaps those who did not get chemotherapy might choose CAM, which they may view as natural and safer over chemotherapy, which, at first, may cause anxiety in some patients. These patients might not be aware that "safe" is not always equivalent to "natural." Additionally, patients may view alternative practitioners as compassionate and invested in the patient's overall well-being, while oncologists typically focus their patient discussions on aspects of their condition [21].

Limitations

The limitations of this study should be acknowledged. First, the sample size is modest and relates to a single geographical region in Saudi Arabia, primarily Jeddah. Thus, the generalization of our findings to other regions may be cautiously taken. Second, the study relies on self-reported data, susceptible to response and recall bias, particularly when assessing CAM use. Furthermore, the study does not explore in-depth the potential interactions between different CAM types and conventional cancer treatments, and it lacks follow-up data to assess the long-term effects of CAM use on cancer outcomes. Additionally, the study's findings may not capture the experiences of those who did not survive or were not diagnosed with BC, potentially introducing selection bias.

## Conclusions

In conclusion, this study highlights the prevalence and motivations behind CAM use among BC survivors in Saudi Arabia. The findings underscore the importance of understanding and integrating CAM into cancer care plans while emphasizing the need for further research to explore the safety and efficacy of specific CAM modalities. These insights have implications for healthcare providers aiming to address patient needs and ensure well-informed decision-making in BC survivorship. In addition, healthcare professionals should be knowledgeable about CAM to facilitate open discussions with patients and integrate CAM use safely into cancer care plans. Larger and more diverse studies are needed to represent the Saudi Arabian population better and provide a comprehensive understanding of CAM utilization among BC survivors.
